# A Sixty-Nine-Year-Old Female With Serratia marcescens Infection

**DOI:** 10.7759/cureus.49985

**Published:** 2023-12-05

**Authors:** Furkhan Ali, JinJin Wu, Dilip KC

**Affiliations:** 1 Internal Medicine, Mount Sinai Hospital, Chicago, USA

**Keywords:** susceptibility, immune compromise, opportunistic bacterial infection, sirs criteria, serratia marcescens

## Abstract

*Serratia marcescens* is a bacterial pathogen that tends to cause opportunistic infections, mainly in immune-compromised patients. In this case, we present a 69-year-old female who presented to the emergency department (ED) at Mount Sinai Hospital in Chicago on February 11th, 2022, after a mechanical fall. She had a medical history of metastatic lung cancer treated with chemotherapy, hypertension, pulmonary embolism, polysubstance abuse, and chronic obstructive pulmonary disease (COPD). The patient was found to be positive for systemic inflammatory response syndrome (SIRS). The patient was then started on broad-spectrum antibiotics, including vancomycin and cefepime. Blood cultures were ordered and came back positive for *Serratia marcescens*. This patient had multiple factors for immune suppression, including metastatic lung cancer, chemotherapy, and polysubstance abuse. The infectious disease department was consulted for the blood culture results, and ceftriaxone was recommended. Later on, levofloxacin was recommended. Blood cultures were negative two days after being positive. Afterward, the patient was kept for monitoring until discharge. *Serratia marcescens* is found in several reservoirs in nature. Therefore, preventing contact with this pathogen in immune-compromised patients can be difficult. It is important to have a degree of clinical suspicion for opportunistic pathogens like *Serratia marcescens* whenever a patient with factors for immune compromise presents for any condition.

## Introduction

*Serratia marcescens* is a relatively rare bacterial infection. It mostly presents as an opportunistic infection [1-7]. Therefore, it more commonly causes infection in some form of the immune-compromised setting. Although multiple *Serratia* species can cause opportunistic infections, *Serratia marcescens* tends to be the most common one [2]. In addition, *Serratia* species are known to have resistance to multiple antibiotics [3,8-10]. In particular, *Serratia* species have intrinsic resistance to ampicillin, amoxicillin, ampicillin-sulbactam, amoxicillin-clavulanate, narrow-spectrum cephalosporins (including cefazolin, cefotaxime, and ceftazidime), cephamycins, cefuroxime, macrolides, tetracycline, and nitrofurantoin, and colistin [3,6]. Furthermore, resistant strains can transfer resistance to strains that are not intrinsically resistant; this can be done through the transfer of R-factors, which are certain kinds of plasmids [4]. This resistance can result in difficulty treating *Serratia* infections. Nevertheless, treatment options are available and based on susceptibility. Sometimes, multiple antibiotics need to be part of the treatment regimen in order to be effective. In this case, we present a 69-year-old female who was brought in for a mechanical fall and found to have a blood infection with *Serratia marcescens*.

## Case presentation

A 69-year-old female with a history of metastatic lung cancer being treated with chemotherapy using erlotinib and complicated by metastases to the brain and bone, hypertension, pulmonary embolism, smoking, IV heroin abuse, and chronic obstructive pulmonary disease (COPD) was brought to the emergency department (ED) at Mount Sinai Hospital in Chicago on February 11th, 2022. She was brought for a mechanical fall while tripping over an oxygen tank at the oncology clinic. The patient was on erlotinib for her chemotherapy. In addition, the patient had a history of heroin and cocaine abuse. Upon arrival at the ED, her vitals were significant for having a temperature of 37, a heart rate of 137, and a respiratory rate of 28. As a result, the patient tested positive for systemic inflammatory response system (SIRS), and thus blood cultures were taken. 

**Figure 1:**
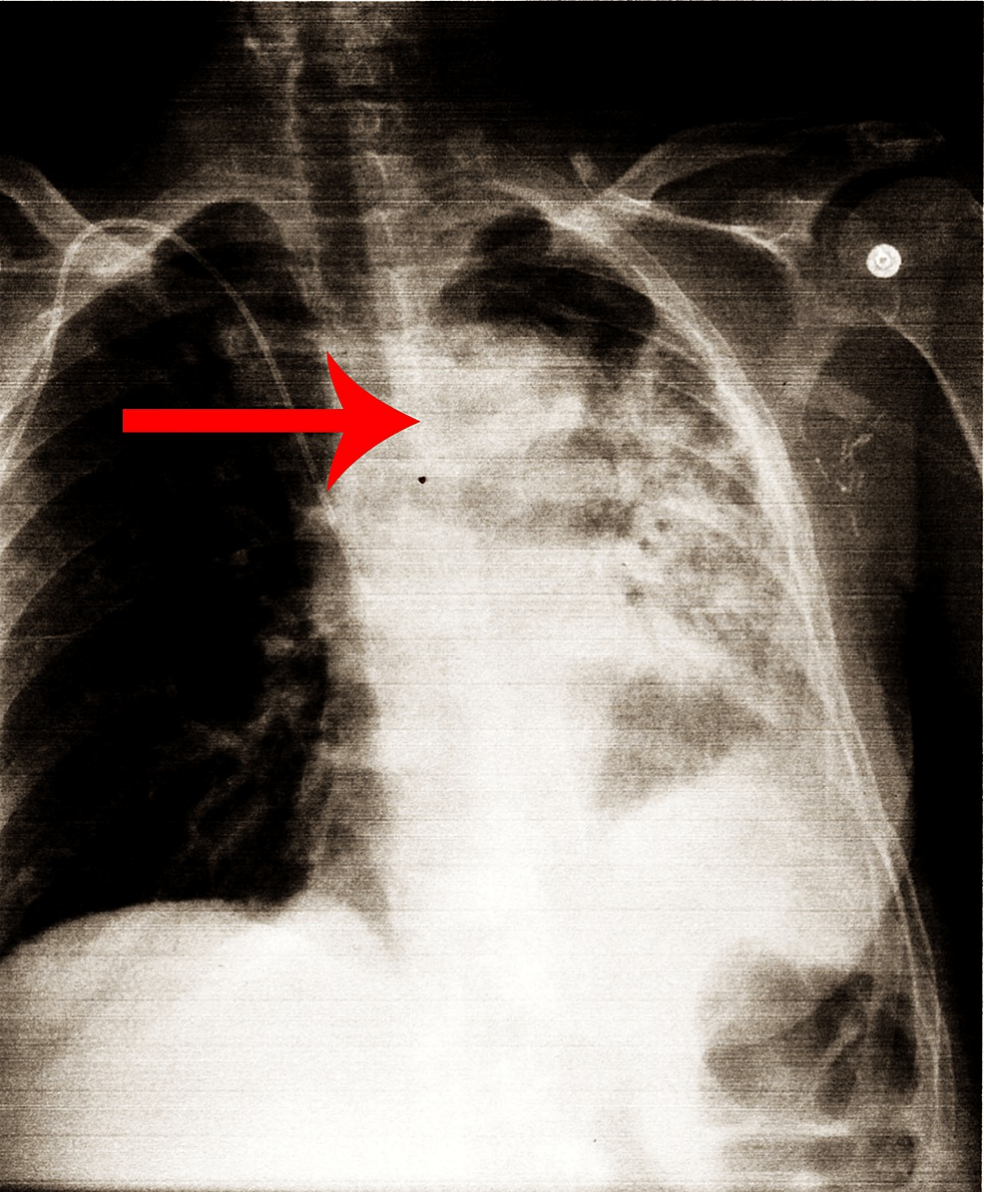
Chest X-ray showing changes near left third rib

Furthermore, she was started on empiric vancomycin and cefepime. The patient was given IV fluids and potassium in the ED. In addition, the patient was found to have hypomagnesemia. Imaging, including chest X-ray, head CT, and echocardiogram (ECHO), were performed. Her brain CT showed no acute processes due to her fall. ECHO did not show any findings concerning endocarditis. Chest X-ray showed changes in the third left rib (Figure 1).

Blood cultures came back positive for *Serratia marcescens. *On the second day of admission, a chest ultrasound showed a small left effusion with atelectasis/consolidation. Repeat blood cultures on February 13th, 2022, came back negative. However, the patient was continued on antibiotics.

Furthermore, the patient endorsed left rib pain. The patient was given pain management via acetaminophen and morphine sulfate, which controlled the pain. Further, the patient complained of abdominal pain. Therefore, an abdominal CT scan was done, which showed no abscesses. 

The infectious disease department was also consulted and recommended switching the patient to ceftriaxone. The patient was then switched to ceftriaxone on February 15th, 2022. Afterward, she was switched to levofloxacin on February 16th, 2022, which was last given on February 18th, 2022. All in all, the patient was on antibiotics for one week. The patient was then kept for monitoring for a few more days. The patient was finally discharged on February 21st, 2022. The patient's course summary is included in Table 1. 

**Table 1:** Summary of the patient admission

Day of admission	Events
1	The patient was admitted to the emergency room for a mechanical fall; image studies did not reveal acute injuries.
1	The patient developed fever, tachycardia, hypotension, and leukocytosis concerning sepsis. The provider decided to pan-culture, including blood culture, urine culture, and sputum culture, to search for an infection source. However, a sputum culture was not sent as the patient did not have sputum. Meanwhile, the patient was given empirical antibiotics, vancomycin and cefepime, and fluid resuscitation, which improved the patient's vital signs.
4	Blood culture came positive for Serratia marcescens. She was susceptible to all the antibiotics. Urine culture was negative. The infection disease physician recommended switching antibiotics to ceftriaxone per sensitive test results.
5	The patient's general condition normalized after antibiotics therapy; a repeat blood culture from 02/13/2022 remained negative for three days.
5	The infection disease physician recommended switching to levofloxacin po 500mg, Q24hrs (02/16-02/18) for better compliance when the patient is discharged home.
10	The patient's general condition is good, and she is discharged home.

## Discussion

*Serratia marcescens* is an opportunistic pathogen with several reservoirs. It can be found in water, soil, plants, and animals [3,4,8]. In addition, *Serratia marcescens* can colonize patients' gastrointestinal or respiratory tract [11]. Colonized individuals may or may not experience symptoms [11]. Nevertheless, it rarely is a cause of primary infection. Rather, it tends to cause opportunistic infections in immune-compromised individuals [1,3-5]. Its abundance in the environment can make transmission difficult to avoid in immune-compromised patients. In this case, transmission can lead to infections such as bacteremia [12]. This patient had several factors contributing to immune suppression. One factor was that this patient had metastatic cancer and was treated with chemotherapy using erlotinib. Chemotherapy can cause immune suppression by inhibiting the production and proliferation of immune cells. This makes chemotherapy patients particularly prone to infection by pathogens that would not affect regular individuals.

Furthermore, cancer can affect an individual's ability to fight infection. Cancer disrupts the body's natural processes, especially when it metastasizes. Cancer cells take up vital nutrients, which deprives healthy cells. In addition, metastases can disrupt surrounding tissues, thus inhibiting them from carrying out their function. Furthermore, many tumors cause cachexia via factors they secrete [13]. This can also decrease a patient's ability to fight infection.

Another factor for immune suppression this patient had was polysubstance abuse. Substance abuse increases the risk of infection [14]. This occurs by contaminating substance delivery sites, especially IV drug injection sites. This allows pathogens to bypass barriers like the skin and enter more vulnerable sites like the blood. Moreover, another risk factor this patient had was old age, which has been associated with an increased risk for *Serratia* infections [15].

Furthermore, there are many other factors which may cause immune suppression. Factors such as diabetes, chronic kidney disease, malnutrition, autoimmune disease medications, steroids, organ transplant rejection prevention medications, hyposplenia, asplenia, human immunodeficiency virus (HIV), and some genetic disorders can all contribute to an immune-compromised state [16]. Scores of patients worldwide suffer from one or more of these conditions. Therefore, they are all at increased risk for opportunistic pathogens such as *Serratia marcescens*. Other examples of opportunistic pathogens are in the table below (Table 2). 

**Table 2:** Opportunistic pathogens

Organism	Source	Presentation(s)
Candida albicans [10]	Oral cavity and genital organs [10]	Endocarditis, oral thrush, urinary tract infection, endophthalmitis [11]
Staphylococcus aureus	Skin [10]	Sepsis [10]
Pseudomonas aeruginosa	Medical devices [10]	Otitis externa, burn infections, pneumonia [10]
Clostridium difficile	Gastrointestinal tract [10]	Diarrhea, colitis [12]
Legionella pneumophila	Water [13]	Pneumonia [10]
Serratia marcescens	Water, soil, plants, and animals [3]	Urinary tract infections, bloodstream infections, and bloodstream infections [13]

Antibiotic treatments for *Serratia *often depend on the infection's severity and sensitivities. Uncomplicated infections, including uncomplicated bacteremia, can be treated with fluoroquinolones, trimethoprim-sulfamethoxazole, piperacillin-tazobactam, third- and fourth-generation cephalosporins, and carbapenems as long as the strain is susceptible [3]. Even in panresistant strains, combining antibiotics can synergistically control *Serratia marcescens* infections [17]. In this case, the ID department recommended cefepime initially despite the patient having sepsis. The patient's condition quickly improved, and she had no evidence of severe hypoxia, acute organ failure, altered mental status, or central nervous system (CNS) infection.

Moreover, her blood culture showed susceptibility to all antibiotics. That is why we narrowed the antibiotics to ceftriaxone per culture results. The patient's general condition improved. Furthermore, repeated blood cultures remained negative, and the patient would be discharged home soon. Therefore, ID recommended switching to the oral antibiotic levofloxacin. 

## Conclusions

Taking care of patients with factors for immune suppression can be a complex task. They are at risk for infection with various opportunistic pathogens. In addition, there is great overlap in the clinical presentations of opportunistic infections. Due to their immune-compromised state, infection with these pathogens can have dire consequences for these patients. Therefore, it is important to have some degree of clinical suspicion for opportunistic infections, such as *Serratia marcescens*, whenever a patient with immune compromising factors presents, even if the chief complaint is not related to infection as in this case.
